# Assessing Decision Fatigue in General Practitioners’ Prescribing Decisions Using the Australian BEACH Data Set

**DOI:** 10.1177/0272989X241263823

**Published:** 2024-07-26

**Authors:** Mona Maier, Daniel Powell, Christopher Harrison, Julie Gordon, Peter Murchie, Julia L. Allan

**Affiliations:** Health Psychology, Institute of Applied Health Sciences, University of Aberdeen, Aberdeen, UK; Health Psychology, Institute of Applied Health Sciences, University of Aberdeen, Aberdeen, UK; School of Public Health, University of Sydney, Sydney, Australia; School of Health Sciences, University of Sydney, Sydney, Australia; Academic Primary Care, Institute of Applied Health Sciences, University of Aberdeen, Aberdeen, UK; Division of Psychology, University of Stirling, Stirling, UK

**Keywords:** decision fatigue, drug prescribing, general practitioners, clinical decision-making, practice patterns, physicians

## Abstract

**Background:**

General practitioners (GPs) make numerous care decisions throughout their workdays. Extended periods of decision making can result in decision fatigue, a gradual shift toward decisions that are less cognitively effortful. This study examines whether observed patterns in GPs’ prescribing decisions are consistent with the decision fatigue phenomenon. We hypothesized that the likelihood of prescribing frequently overprescribed medications (antibiotics, benzodiazepines, opioids; less effortful to prescribe) will increase and the likelihood of prescribing frequently underprescribed medications (statins, osteoporosis medications; more effortful to prescribe) will decrease over the workday.

**Methods:**

This study used nationally representative primary care data on GP-patient encounters from the Bettering the Evaluation and Care of Health program from Australia. The association between prescribing decisions and order of patient encounters over a GP’s workday was assessed with generalized linear mixed models accounting for clustering and adjusting for patient, provider, and encounter characteristics.

**Results:**

Among 262,456 encounters recorded by 2,909 GPs, the odds of prescribing antibiotics significantly increased by 8.7% with 15 additional patient encounters (odds ratio [OR] = 1.087; confidence interval [CI] = 1.059–1.116). The odds of prescribing decreased significantly with 15 additional patient encounters by 6.3% for benzodiazepines (OR = 0.937; CI = 0.893–0.983), 21.9% for statins (OR = 0.791; CI = 0.753–0.831), and 25.0% for osteoporosis medications (OR = 0.750; CI = 0.690–0.814). No significant effects were observed for opioids. All findings were replicated in confirmatory analyses except the effect of benzodiazepines.

**Conclusions:**

GPs were increasingly likely to prescribe antibiotics and were less likely to prescribe statins and osteoporosis medications as the workday wore on, which was consistent with decision fatigue. There was no convincing evidence of decision fatigue effects in the prescribing of opioids or benzodiazepines. These findings establish decision fatigue as a promising target for optimizing prescribing behavior.

**Highlights:**

- We found that as general practitioners progress through their workday, they become more likely to prescribe antibiotics that are reportedly overprescribed and less likely to prescribe statins and osteoporosis medications that are reportedly underprescribed.
- This change in decision making over time is consistent with the decision fatigue phenomenon. Decision fatigue occurs when we make many decisions without taking a rest break. As we make those decisions, we become gradually more likely to make decisions that are less difficult.
- The findings of this study show that decision fatigue is a possible target for improving guideline-compliant prescribing of pharmacologic medications.

Over the course of a working day, general practitioners (GPs) make a series of decisions that affect patient outcomes. Rational models suggest that important decisions, such as those about whether to prescribe medications, are solely based on a balanced assessment of the available information. However, human decision making is susceptible to bias,^1^ and suboptimal decisions are made relatively frequently in the health care context.^2^ For example, while all medications are subject to prescribing guidelines, some are commonly overprescribed while others remain underused,^3,4^ and there is some evidence that prescribing changes over the course of the working day.^5–7^ One potential reason for this is decision fatigue, a systematic shift that occurs in decision making as the time spent on tasks increases.^8^ The aim of this study was to examine whether observed patterns in Australian GPs’ prescribing decisions over the working day are consistent with the decision fatigue phenomenon. In Australia, about 87% of people will visit a GP at least once a year, and in 2015–2016, there were 6 GP visits per capita, on average.^9^ General practice visits are subsidized on a fee-for-service model through Medicare, Australia’s universal health care system,^9^ and for most visits, the patient does not pay any out-of-pocket cost.^10^

Studies have shown that health care decision making changes systematically over the course of a workday.^8^ For example, studies have shown that surgeons were less likely to decide that patients need surgery toward the end of workdays,^11^ triage nurses working at a medical telephone helpline became increasingly likely to make conservative triage decisions as time since their last break increased,^12^ and doctors from a range of specialities became less likely to order prostate cancer screening tests for patients attending outpatient clinics as the day wore on.^13^ One explanation for this systematic change is decision fatigue, a tendency to gradually shift toward making decisions that are cognitively less effortful as the time spent working, or the number of consecutive decisions made without a break, increases.^14^ Decision fatigue is a general phenomenon that has been observed across a diverse range of contexts in which decisions are made sequentially over time. For example, court judges became progressively less likely to approve parole requests (in favor of keeping the prisoner incarcerated) as court sessions wore on^15^ (this study by Danziger et al.^15^ has received considerable critique for overestimation of effect sizes, and the results should be interpreted cautiously^16–18^), credit officers in the finance sector were less likely to approve credit loans during the midday period compared with early in the workday,^19^ and academic journal editors rejected proportionally more manuscripts when reviewing larger numbers of papers.^20^ In each case, decision making changes systematically over the work period as the number of decisions increases, and in particular as the number of decisions made without a break increases.

As workers in health care settings regularly work lengthy periods without a rest break,^21,22^ decision fatigue may be particularly pronounced in this context. Recent surveys from the United Kingdom show that GPs are “experiencing unprecedented workload demands,”^22^ that the majority (77%) take no breaks during 4-h clinic sessions, and that more than a quarter (28%) work full days without a proper break.^21^ This does not appear to be a problem that is specific to the United Kingdom.^23^ Working continuously for unbroken periods provides an ideal opportunity for decision fatigue to develop, with clear implications for patient care and resource allocation.^24^ In line with this, existing evidence suggests that GPs appear to make different decisions over the work period, for example, delivering fewer flu vaccinations later in the day^25^ and becoming less likely to order cancer screening for their patients over the course of a clinic session.^26^ Pharmacologic prescribing decisions also appear to change, with GPs issuing a higher number of clinically conservative but potentially unnecessary prescriptions for antibiotics and opioids over the course of a workday^5,6,27,28^ and prescribing fewer potentially beneficial but often underprescribed statins.^29^ To date, however, these opposing patterns of increasing and decreasing prescribing likelihoods have not been directly contrasted, and there is a lack of discussion around why decision fatigue effects are different for decisions about different medications. For typically overprescribed medications (such as antibiotics or opioids, which are often prescribed reactively in response to acute symptoms), prescribing (compared with not prescribing) is in many cases likely to be the default decision. Selecting the default is cognitively easier, as prescribing reduces the need to explain alternative approaches to symptom management and often meets perceived demand from patients.^30^ For typically underprescribed medications (such as statins, which are often prescribed preventively to ward off future health problems), we assume that it is cognitively easier to not prescribe, as nonprescribing is the default decision for these medication types. Preventive prescribing likely involves additional cognitive effort as preventive medications may not come to mind automatically during consultations about acute health problems, and prescribing may require the GP to review previous diagnostic test results to ascertain suitability and/or persuade patients of future benefits of the medication in the face of potential side effects.

Inappropriate prescribing of medications is important to understand as it can have negative consequences for patients, the health care system, and society.^31^ For example, overuse of antimicrobials (including antibiotics) is a key driver in the development of medication-resistant pathogens,^32^ posing a significant threat to wider society.^33^ Prolonged use of prescribed opioids and benzodiazepines can lead to addiction and dependence, with associated harms that are an issue of great public health interest internationally.^9,10^ Conversely, underprescribing of medications can also be detrimental to population health. For example, while guideline-directed statin use has been shown to reduce the risk of major adverse cardiovascular events, there are significant shortfalls in preventive prescribing.^30^ Similarly, effective medications for osteoporosis help to prevent further bone fractures in patients with the condition; however, they are not prescribed to almost one-quarter of patients with osteoporosis.^34^

This study aimed to systematically investigate patterns in GPs’ pharmacologic prescribing decisions about multiple medications over the working day, using a large, nationally representative Australian general practice data set. In contrast to existing studies, we accounted for and reported variances between different GPs. To test whether observed patterns were consistent with decision fatigue, we selected a variety of medications, some of which are reportedly overprescribed and may be directly requested by patients to resolve immediate and acute symptoms (opioids, antibiotics, benzodiazepines^35–37^) and some that are reportedly underprescribed and may require patients to be persuaded to take them to prevent possible future adverse outcomes with no immediate benefit (statins, osteoporosis medications^30^). We tested the following decision fatigue hypotheses: over the course of the working day, as the number of decisions made by GPs increases, the odds of selecting the less effortful default option will increase, and so,

1. the odds of prescribing reportedly overprescribed medications (antibiotics, opioids, benzodiazepines) will increase and
2. the odds of prescribing reportedly underprescribed preventive medications (statins, osteoporosis medications) will decrease.

## Methods

This retrospective cohort study used data on general practice encounters recorded by a representative sample of GPs across Australia from the Bettering the Evaluation and Care of Health (BEACH) study.^38^ BEACH was a cross-sectional, national study of GP clinical activity in Australia that ran between 1998 and 2016. Every year, a national, rolling, random sample of approximately 1,000 GPs provided information about 100 consecutive encounters with consenting, unidentified patients. As patients remained unidentified, the same patient may attend multiple encounters within the sample, so we refer to “patient encounters” throughout rather than “patients.” Participating GPs also supplied information about themselves and their practice.

The sample size for this study was determined pragmatically from the number of physician-patient encounters available for inclusion within the data set. The complete BEACH data set covers 18 years with more than 1.7 million representative GP-patient encounters. The first 2 BEACH years were excluded (203,100 GP-patient encounters), as some variables of interest were collected only from the year 2000 onward. As our primary interest in decision fatigue required a sequence of decisions, we excluded encounters on workdays on which GPs recorded fewer than 10 encounters (150,952 GP-patient encounters). This is in keeping with a previous study of prescribing trends throughout the clinic day.^28^ Due to computational limitations when running our preferred statistical approach (see below) with such a large data set, our hypotheses were tested in a 3-year BEACH data subset: the most recent period, 2013–2016 (262,456 GP-patient encounters). A second randomly selected alternative time period (2000–2003, 271,519 GP-patient encounters) was also analyzed for confirmatory purposes.

### Statistical Analysis

For all analyses, we used mixed-effects logistic regression models to model the odds of prescribing different categories of medications as a function of decision fatigue, controlling for the nesting of multiple patient encounters within the same GP. For each patient encounter, we generated a prescription outcome as a binary variable indicating whether a target medication was (1) or was not (0) prescribed. Target medications were those categorized as 1) antibiotics, 2) opioids, 3) benzodiazepines, 4) statins, or 5) osteoporosis medications. The Anatomical Therapeutic Chemical codes used to classify medications within each target category are listed in the supplementary materials (Supplementary Table S1). We operationalized decision fatigue within the data as a variable representing the sequential order of each patient encounter within the series of all encounters completed by a particular GP during a working day (i.e., the first consultation by each GP on a given date was classified as 1, the next as 2 and so on, numbering all following encounters). The method of using order number of encounter as a proxy for decision fatigue has been established in previous studies.^11,12^

In all analyses, we adjusted for key characteristics of patients, physicians, and encounters that may influence decision making. Patient characteristics included in our models were age, sex, indigenous status, Commonwealth Health Care Card status, and new or returning patient. Physician characteristics were age, sex, and rurality of practice. Encounter characteristics were the weekday, season, and year.

Adjusted 2-level mixed-effects logistic regression models for dichotomous outcomes were used for all outcome variables, to account for the clustering of encounters within physicians. All models used the logit link such that reported model fixed-effect estimates can be interpreted as the increased odds of prescribing for every 1-unit increase in the predictor. Multivariable models adjusted for physician-level factors and encounter-level factors (i.e., patient and encounter characteristics) and included the decision fatigue effect (encounter order number) as the main effect of interest. All models sought to include the maximal random effects structure supported by the data^39^ using an unstructured variance-covariance matrix to permit variance in the intercept, slope (decision fatigue effect), and their covariance. Modeling of covariance was dropped where covariance could not be achieved. As recommended in analyses of intensive longitudinal data,^40^ models accounted for the autocorrelation of errors with an autoregressive error matrix.

For all analyses, we present adjusted point estimates (odds ratios; OR) with associated 95% confidence interval (CI) and *P* value. Two-sided hypothesis tests used a significance level of 0.05, and all analyses were conducted using PROC GLIMMIX in SAS version 9.4 (SAS Institute Inc.).

A sensitivity analysis using conditional logistic regression modeling^41^ is reported in the supplementary material (S3.1–S3.5) to check results were not dependent on the choice of analysis. In addition, we present conditional logistic regression models using dummy variables reflecting groups of encounters as categories (1–5, 6–10, 11–15, 16–20, 21–25, 26–30, 31–35, 36–40, 41+) to examine potential nonlinear effects, in the supplement (S4.1–S4.5).

## Results

### Study Population

The BEACH data set from 2013–2016 includes records for 291,900 GP-patient encounters from 2,919 GPs participants. For the present study, 29,444 patient encounters were excluded as they were on days for which the GP recorded fewer than 10 encounters. This led to a study sample of 262,456 GP-patient encounters recorded by 2,909 GPs. The confirmatory analyses subset from 2000–2003 includes records from 234,219 GP-patient encounters from 2,857 GPs participants.

Table 1 provides the summary statistics for the study sample (2013-2016). The mean number of encounters per workday was 15.27. Nearly 60% of GP-patient encounters were with male GPs, more than 45% of encounters were with GPs aged 55 y or older, two-thirds of encounters were with GPs who had graduated from medical school in Australia, and more than 70% of encounters were with GPs who worked in major cities. Half of encounters included patients aged between 25 and 64 y old, and more than a third of encounters were with patients aged 65 y or older. Most encounters (>90%) included patients who had been seen previously by the participating GP, more than 90% of patients at encounters were from an English-speaking background, and 40% of encounters were with male patients.

**Table 1:** Sample Characteristics of General Practitioners and Patients at Recorded Encounters^a^

Variable	Descriptive Statistics	
	*n*	% (95% CI)
Encounters by GP characteristics		
GP-patient encounters	262,456	—
Sex		
Female	110,745	42.19 (40.38–44.01)
Male	151,711	57.80 (55.99–59.62)
Age, y		
<45	68,712	26.33 (24.70–27.95)
45–54	71,205	27.28 (25.63–28.93)
55+	121,084	46.39 (44.55–48.24)
Graduated from medical school in Australia		
Yes	174,708	66.85 (65.12–68.59)
No	86,626	33.15 (31.41–34.88)
Rurality		
Major city	184,725	70.54 (68.86–72.22)
Inner regional	53,229	20.33 (18.84–21.81)
Outer regional/remote	23,906	9.13 (8.07–10.19)
Encounters by workday characteristics		
Total number of workdays	19,105	—
Mean number of patient encounters per workday	15.27	15.14–15.41
BEACH year		
2014	86,487	32.95 (31.22–34.69)
2015	89,476	34.09 (32.34–35.84)
2016	86,493	32.96 (31.22–34.69)
Weekday		
Monday	50,399	19.20 (18.60–19.81)
Tuesday	62,053	23.64 (23.06–24.23)
Wednesday	54,788	20.88 (20.30–21.45)
Thursday	48,840	18.61 (18.07–19.14)
Friday	37,873	14.43 (13.90–14.96)
Saturday	6,337	2.41 (2.14–2.69)
Sunday	2,166	0.83 (0.65–0.10)
Season^b^		
Spring (September–November)	67,116	25.57 (24.0–27.15)
Summer (December–February)	58,134	22.15 (20.66–23.64)
Autumn (March–May)	73,879	28.15 (26.52–29.78)
Winter (June–August)	63,327	24.13 (22.58–25.68)
Encounters by patient characteristics		
Sex		
Female	154,124	59.25 (58.72–59.78)
Male	105,989	40.75 (40.22–41.28)
Age, y		
0–4	16,101	6.19 (5.97–6.40)
5–14	13,287	5.11 (4.95–5.26)
15–24	20,347	7.82 (7.60–8.04)
25–44	56,629	21.76 (21.33–22.20)
45–64	70,421	27.07 (26.75–27.38)
65–84	68,338	26.26 (25.74–26.79)
85+	15,068	5.79 (5.54–6.05)
Type of visit		
New to practice	17,759	93.14 (92.82–93.45)
Seen previously	241,026	6.86 (6.55–7.18)
Health care/benefits card status		
Holder	109,568	45.50 (44.68–46.33)
Nonholder	131,236	54.50 (53.67–55.32)
Non–English-speaking background		
Yes	20,794	8.76 (8.06–9.47)
No	216,467	91.24 (90.53–91.94)
Socioeconomic index		
High level of advantage in area	154,086	60.00 (58.59–61.43)
Low level of advantage in area	102,690	39.99 (38.57–41.41)
Indigenous status		
Indigenous	3,956	1.67 (1.44–1.90)
Nonindigenous	233,255	98.33 (98.10–98.56)

CI, confidence interval; GP, general practitioner.

a Values are numbers (percentages) unless otherwise stated. Where frequencies do not total to the complete number of GP-patient encounters, this is due to missing data.

b Using the meteorological calendar for Australia.

Table 2 displays the total number of encounters for the study sample (2013–2016) at which the targeted medications were prescribed and the prescribing rates with 95% CIs. Antibiotics were the most frequently prescribed, at 12.13% of recorded encounters. Prescribing rates of benzodiazepines, opioids, statins, and osteoporosis medications ranged between 1.24% and 5.31%.

**Table 2:** Frequency and Proportion of Recorded Encounters in Which a (Specific) Pharmacologic Prescription Was Recorded

Dependent Variable	Total Prescriptions (*n*)	Prescribing Rate (95% CI)
Antibiotic prescription	31,843	12.13% (11.88–12.38)
Opioid prescription	13,925	5.31% (5.15–5.46)
Benzodiazepine prescription	7,733	2.95% (2.83–3.06)
Statin prescription	9,409	3.58% (3.47–3.70)
Osteoporosis medication prescription	3,250	1.24% (1.18–1.30)

CI, confidence interval.

### Appointment Order Number (Decision Fatigue) and Prescribing Decisions

**Figure 1:**
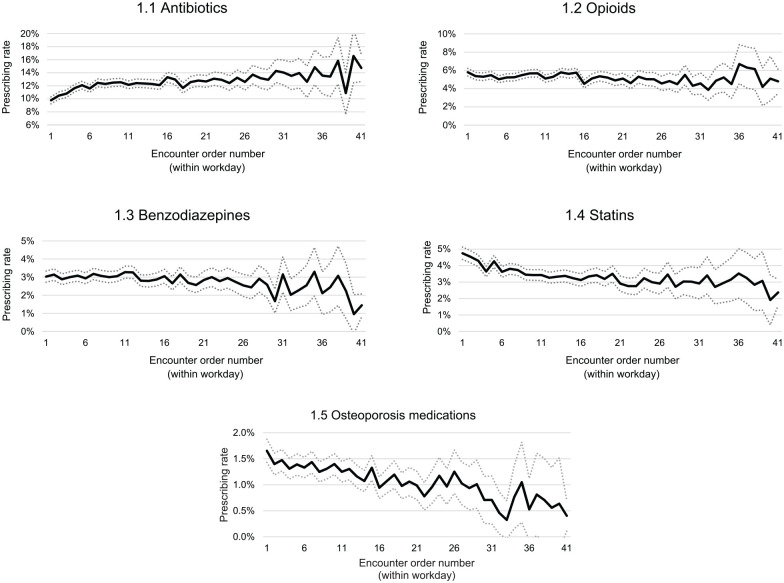
Unadjusted prescribing rates of medications by encounter position within the workday for all general practitioners. Encounters with a position of 41 and over were grouped due to low numbers. Dashed lines represent 95% confidence intervals at each encounter position.

Figure 1 displays the unadjusted prescribing rates of the target medications (antibiotics, benzodiazepines, opioids, statins, and osteoporosis medications), by encounter position within the workday for all GPs. Encounters with a position of 41 and over were grouped due to low numbers. The graphs show that the rate of antibiotic prescriptions increased as the workday progressed (Figure 1.1) and the rates of prescribing for statins (Figure 1.4) and osteoporosis medication prescriptions (Figure 1.5) decreased. Ten percent of patient encounters at encounter position 1 (the start of a GP’s workday) had an antibiotic prescription recorded, and this increased to 16.6% for patient encounters at the 40th position within GPs’ workday. For the first encounter of the workday, the prescribing rate for statins was 4.7%, more than double the prescribing rate at position 40 (1.9%), and the prescribing rate for osteoporosis medications was at 1.7% at the first encounter and dropped to 0.4% at the 40th encounter. For benzodiazepines, the trend is less clear, but a visual inspection of the CIs shows that there is a decrease in prescribing rates (Figure 1.3). Opioid-prescribing rates showed no clear trends on visual inspection (Figure 1.2).

Results from the main adjusted 2-level mixed-effects logistic regression models (using the 2013–2016 study sample) are presented in Table 3. The intercept OR corresponds to the estimated prescribing rate before the very first encounter in a workday, when all variables are set to zero. The main fixed effect is the estimated effect of an additional patient encounter on the odds of a GP prescribing a certain medication. Random effect and covariance parameter estimates are listed in the rows below.

**Table 3:** Effect of Ordinal Encounter Position on the Odds of Prescribing (Specific) Pharmacologic Medications in Adjusted 2-Level Mixed-Effects Logistic Regression Models (Study Sample 2013–2016)^a^

Dependent Variable: Prescription Recorded (1 = Yes, 0 = No)	Antibiotics	Opioids	Benzodiazepines	Statins	Osteoporosis Medications
Fixed effects^b^					
Intercept	OR = 0.112***	OR = 0.051***	OR = 0.013***	OR = 0.036***	OR = 0.009***
	0.099–0.126	0.044–0.059	0.010–0.016	0.030–0.044	0.007–0.012
Encounter’s ordinal position effect	OR = 1.006***	OR = 0.998	OR = 0.996**	OR = 0.984***	OR = 0.981***
	1.004–1.007	0.996–1.001	0.992–0.999	0.981–0.988	0.976–0.986
Random effects					
Level 2 (between-person)					
Variance in intercept	0.532***	0.303***	0.834***	0.309***	1.192***
	0.017	0.016	0.027	0.018	0.036
Variance in effect	0.010***	<0.001	0.025***	0.001***	0.057***
	0.002	0	0.002	0.001	0.002
Covariance	−0.005***	N/A	−0.013***	N/A	−0.048***
	0.001	—	0.002	—	0.004
Level 1 (within-person)					
Residual	0.942***	0.901***	0.752***	0.721***	0.530***
	0.003	0.003	0.002	0.002	0.002
Autocorrelation	0.024***	0.008***	0.005*	0.007***	0
	0.002	0.008	0.002	0.002	0.002

a Fixed covariates (general practitioner [GP] sex, GP age, rurality, year, weekday, season, patient sex, patient age, type of visit, health care/benefits card status, non–English-speaking background, socioeconomic index, indigenous status) were omitted from the table for parsimony but are reported in full in the supplementary material (S2.1–S2.5).

b Fixed effects: odds ratios (ORs) with 95% confidence intervals (CIs) below. Random effects: (co-)variance parameter estimates, with standard errors below; where covariance is N/A, this was omitted to facilitate model convergence; residual estimates.

* *P* < 0.05; **P < 0.01; ***P < 0.001.

In line with our first hypothesis, the odds of prescribing antibiotics significantly increased as GPs progressed through their workday and therefore made more decisions, increasing by 0.6% on average with every additional patient encounter (OR = 1.006, *P* < 0.01, 95% CI [1.004, 1.007]). However, the odds of prescribing benzodiazepines reduced, on average, by 0.4% with each patient encounter (OR = 0.996, *P* < 0.01; 95% CI [0.992, 0.999]), and there was no evidence of any systematic change over the sequence of encounters for opioid prescriptions (OR = 0.998, *P* = 0.13; 95% CI [0.996, 1.001]). In line with our second hypothesis, both the odds of prescribing statins and osteoporosis medications significantly decreased as GPs progressed through their workday and therefore made more decisions: the odds of statin prescriptions decreased by 1.6% with each encounter (OR = 0.984, *P* < 0.01; 95% CI [0.981, 0.988]), and the odds of prescribing osteoporosis medication prescriptions decreased by 1.9% (OR = 0.981, *P* < 0.01; 95% CI [0.976, 0.986]).

While our main focus of interest is the estimated effect of each additional patient encounter on the odds of a GP prescribing a certain medication, rescaling this variable so that a 1-unit change corresponds to the mean number of patient encounters each day (*n* = 15) illustrates the magnitude of the change in prescribing across an average working day. After rescaling, the odds of prescribing antibiotics significantly increased by 8.7% on average with 15 additional patient encounters. The odds of prescribing decreased significantly with 15 additional patient encounters by 6.3% for benzodiazepines, 21.9% for statins, and 25% for osteoporosis medications.

Results of the confirmatory analyses (using the 2000–2003 study sample) are reported in Table 4. Results for antibiotics, opioids, statins, and osteoporosis medications showed very similar main effects of patient encounter order number on the odds of prescribing. In contrast to the unexpected significant decrease in benzodiazepine prescription in the main 2013–2016 study sample, there was no evidence in the confirmatory sample of any systematic change over the sequence of encounters for benzodiazepine prescriptions (OR = 0.999, *P* = 0.33; 95% CI [0.996, 1.001]).

**Table 4:** Effect of Ordinal Encounter Position on Odds of Prescribing (Specific) Pharmacologic Medications in Adjusted 2-Level Mixed-Effects Logistic Regression Models (Study Sample: 2000–2003)^a^

Dependent Variable: Prescription Recorded (1 = Yes, 0 = No)	Antibiotics	Opioids	Benzodiazepines	Statins	Osteoporosis Medications
Fixed effects^b^					
Intercept	OR = 0.116***	OR = 0.034***	OR = 0.021***	OR = 0.017***	OR = 0.003***
	0.102–0.131	0.028–0.028	0.017–0.026	0.013–0.022	0.002–0.005
Encounter’s ordinal position effect	OR = 1.004***	OR = 1.000	OR = 0.999	OR = 0.977***	OR = 0.989***
	1.002–1.005	0.998–1.003	0.996–1.001	0.973–0.980	0.983–0.995
Random effects					
Level 2 (between-person)					
Variance in intercept	0.504***	0.391***	0.738***	0.371***	0.837***
	0.016	0.019	0.025	0.023	0.05
Variance in effect	0.007***	<0.001	0.017***	<0.001***	N/A
	0.002	0	0.002	0	—
Covariance	−0.003*	N/A	−0.008***	N/A	N/A
	0.001	—	0.002	—	—
Level 1 (within-person)					
Residual	0.952***	0.858***	0.863***	0.770***	0.636***
	0.003	0.003	0.003	0.002	0.002
Autocorrelation	0.021***	0.009***	0.010***	0.004	0
	0.002	0.002	0.002	0.002	0.002

a Fixed covariates (general practitioner [GP] sex, GP age, rurality, year, weekday, season, patient sex, patient age, type of visit, health care/benefits card status, non–English-speaking background, socioeconomic index, indigenous status) were omitted from the table for parsimony but are reported in full in the supplementary material (S2.1–S2.5).

b Fixed effects: odds ratios (ORs) with 95% confidence intervals below; Random effects: (co-)variance parameter estimates, with standard errors below; where covariance is N/A, this was omitted to facilitate model convergence; and residual estimates.

* *P* < 0.05; ***P < 0.001.

### Variation between GPs

The random effects estimates of all models indicate that the intercept (initial odds of prescribing) and the encounter order number effects themselves varied significantly across individual GPs (see Tables 3 and 4). For all medications, the intercept variance estimates were statistically significant (*P* < 0.01), meaning that baseline prescribing rates varied significantly between GPs, and some GPs were generally more likely to prescribe than others were. The variance in effect estimate was also statistically significant (*P* < 0.01) for antibiotics, benzodiazepines, and osteoporosis drugs, meaning that the magnitude of the decision fatigue effect varied substantially across the GPs in the sample.

### Sensitivity Analyses Results

The results of the sensitivity analyses showed very similar findings to the main analyses (see S3.1 to S4.5). The conditional logistic regression models with linear predictors for encounter order number (see S3.1 to S3.5) indicated no meaningful differences in the results compared with the mixed-effect models. When modeling encounter order number using dummy variables reflecting groups of encounters as categories (see S4.1 to S4.5), the results indicated that the effect for benzodiazepines was not statistically significant until the 41+ encounter group, whereas the antibiotics effect showed the largest effect between the 1 to 5 and 6 to 10 groups before the increase in prescribing rates slowed.

## Discussion

Among a large nationally representative data set of 262,456 GP-patient encounters recorded by 2,909 GPs in Australia (as well as a confirmatory analyses sample of 234,219 GP-patient encounters recorded by 2,857 GPs), we found behavioral patterns consistent with decision fatigue in GP decision making. GPs’ decisions to prescribe medications varied systematically across the working day: after controlling for known covariates, the odds of prescribing antibiotics increased significantly, whereas the odds of prescribing statins, osteoporosis medications, and benzodiazepines decreased significantly. Opioid prescribing did not change significantly throughout the workday. These findings somewhat reflect the current decision fatigue literature and support the existence of predictable directional effects for antibiotics prescribing^5^ and statin prescribing.^7,29^ Previously shown increases in the prescribing of opioids were not replicated.^6,27,28^ To our knowledge, there have been no previous studies investigating changes in prescribing likelihood for benzodiazepines or osteoporosis medications over the course of a workday.

GPs in the present study became more or less likely to prescribe particular medications over the course of the working day depending on whether the medication in question had a reactive (antibiotics) versus preventive application (statins, osteoporosis medications) and was therefore cognitively easier to prescribe or not prescribe. These findings are in line with our original hypotheses and are consistent with the presence of decision fatigue as more cognitively effortful decisions became less likely as the number of decisions made by GPs increased over the working day. However, the odds of prescribing opioids did not change over the day in the present analysis, in contrast to both our original hypothesis and findings in the existing literature.^6,27,28^ One possible explanation for the lack of change in opioid prescribing over time is that our analyses use Australian data, whereas all prior published studies use data from the United States. In Australia there are stricter opioid-prescribing regulations for health care professionals and restrictions on direct advertising of pharmaceuticals to patients,^42^ which may lower the demand for or expectations about opioids from Australian patients relative to those in the United States. This in combination could mean that prescribing opioids is an exceptional rather than default option in the Australian context, reducing the likelihood that prescribing would be affected by decision fatigue.

Benzodiazepines are mainly prescribed to manage anxiety and insomnia, acute symptoms a patient wishes to have alleviated. Therefore, we expected these prescriptions to increase over the course of the working day. We found no support for this. Indeed, in the 2013–2016 sample, we found these prescriptions decreased. However, this effect should be treated with caution as we found evidence this was driven by a nonlinear effect found at the very end of the longest shifts and that the effect did not replicate in the confirmatory analysis with the alternative time-period sample. Overprescribing of benzodiazepines gained mainstream awareness in the 2010s in relation to the wider opioid crisis, and Australian authorities introduced additional restrictions on benzodiazepine prescriptions, so the absence of the expected effect here may have a similar explanation as that for opioids.

The sensitivity analyses conducted in this study offer valuable insights into the robustness of our findings. Given the absence of a universally accepted method for investigating decision fatigue in observational studies—where some use mixed-effect models^43^ while others rely on conditional logistic regressions^44^—it is reassuring that both our mixed-effect models and conditional logistic regression models yield effects with no substantial differences. Notably, the existing decision fatigue literature exhibits considerable variation in the proxies used as predictors of decision fatigue. Apart from variances in selected measures (such as time into shift or appointment order number), studies differ in using either linear predictors^5,28^ or categorical predictors.^7,44^ Our results suggest that the manifestation of the decision fatigue effect may vary depending on the context. Specifically, for antibiotics, the effect seems to emerge rapidly (within the first 5 encounters) and continues to increase at a slower rate, possibly reflecting a relatively quick decline in GPs’ motivation to persuade a patient they do not require an antibiotic. As already discussed, for benzodiazepines, our nonlinear models indicate that the effect is primarily driven by encounters late in the sequence of encounters (with order numbers of 41 or above). Noting that the small effect for benzodiazepines also did not replicate in the alternative time period, this suggests the small effect we found for this medication is likely an artifact of a significant drop in prescribing rates at the tail end of the very longest shifts. Further research is warranted to elucidate the timing and mechanisms underlying the emergence of the decision fatigue effect.

Our results also suggest that variation in decision fatigue effects across GPs should be considered as our models demonstrate that there is significant variance between physicians in both the initial prescribing likelihood at the start of a workday and in the magnitude of the decision fatigue effect itself, meaning that some GPs demonstrate higher decision fatigue effects than others do. This raises the prospect of potential moderators, which may help to understand these effects further. Potentially modifiable factors such as individual expertise and beliefs about certain medications and health conditions could play a role in prescribing decision making. Furthermore, factors such as workload, burnout, or generally increased levels of decision making due to circumstances in a GP’s personal life could make some individuals more vulnerable to the effects of decision fatigue on prescribing. However, in the sensitivity analysis, we present where fixed effects control for all GP-level factors, there were no meaningful differences in the study results, suggesting that such potential confounders were not influential here.

There are differing theoretical explanations for why decision fatigue arises. Early explanations centered on “ego depletion,” a concept that suggested that any “act of volition” draws on some limited resource, similar to strength or energy. This resource was thought to be reduced with every additional act of volition, eventually becoming depleted, leading to a reduction in an individual’s capacity or willingness to engage in further effortful actions (including making choices and initiating action).^45^ When Pignatiello et al. First formally conceptualized decision fatigue, they suggested that it be considered a “symptom or phenotypic expression of ego depletion.”^14^ However, in recent years, high-quality, multilab preregistered replication studies have demonstrated that any ego depletion effect is likely close to zero, rendering this an unlikely explanation.^46^ Alternative models that could explain predictable effort-related shifts in decision making over time include the process model of depletion, which suggests that exertion of self-control or mental effort reduces motivation to effortfully exert further self-control or effort while shifting attention toward possible sources of gratification and reward, and increasing the likelihood of acting on impulse.^47^ Similarly, the literature on mental fatigue^48^ and vigilance decrement^49^ also shows that time on task leads to cumulative impairments in task performance, attributing changes to a decrease in the efficiency, or availability, of cognitive resources and reporting links between sustained attention and personality traits. To date, little work has been done to disentangle these differing conceptualizations, and more research and theorization are needed to better understand the mechanisms underlying the decision fatigue effect.

The current results suggest that patients seen at different times throughout a GP’s workday have a different likelihood of being prescribed particular medications depending on when their appointments are scheduled. They also suggest that prescribing might become less efficient as the workday wears on, with an increase in potentially unnecessary reactive prescriptions and a decrease in potentially beneficial preventive prescriptions that could reduce the need for more costly treatment later. Medical care in the modern era is becoming increasingly complex: the general population of patients, due to demographic shifts, are older and have more comorbidities than previously, and large numbers of new medications have widened the pool of options to be considered when prescribing. Consequently, prescribing is likely to be more cognitively effortful in general for modern health care professionals, potentially magnifying any underlying decision fatigue effects. It may be possible to reduce decision fatigue and to optimize the efficiency and equity of clinical decisions by implementing appropriate decision support tools and frequent break scheduling. For example, the electronic medical record could be adapted to reduce cognitive demand, for example, by including the option to easily print out lay explanations of why particular medications might (not) be beneficial for a patient, which the GPs can use as a memory aid when addressing common concerns or explaining why a patient’s expectation for prescribing will not be fulfilled. Regularly scheduled (compulsory) breaks ought to be considered, although implementation would inevitably be challenging. Generally overstretched health care systems with limited resources and wide-reaching staff shortages are likely to create an optimal environment for decision fatigue, by creating the need for health care workers to engage in longer periods of work without breaks. These pressures have been further amplified by the COVID-19 pandemic and are unlikely to change without significant policy intervention and additional resource allocation.

Findings from this study can likely be generalized beyond the Australian context as the Australian health care system is broadly comparable to many others around the world. For example, the Australian, Norwegian, Dutch, and the UK health care systems score similarly in terms of global performance rankings (third, first, second, and fourth, respectively),^50^ and many other countries have health care systems that provide universal health care coverage.^51^ Burdens on the system are also relatively comparable. For example, the share of GPs compared with physicians overall has decreased in many countries, specifically in Australia, the United Kingdom, Denmark, Israel, Estonia, and Ireland, by more than 20% between 2000 and 2017,^52^ leading to likely workload increases for GPs working in those countries. Consequently, the present patterns in decision making will be highly likely to generalize to decisions made in other similar health care systems. Different payment structures across and within health care systems could affect decision fatigue effects and require further investigation.

The current study has a number of strengths and limitations. The size and scale of the nationally representative BEACH study compares favorably to previous research. Similarly, simultaneously assessing and comparing the prescribing of 5 different pharmacologic medications with different characteristics and hypothetical responses to decision fatigue strengthens the evidence base. All analyses were adjusted for a wide range of patient, GP, and encounter characteristics to control for possible confounders, and multilevel modeling was used to account for the hierarchical structure of the data and to model both within- and between-person effects. To further account for potential omitted confounders, all analyses included residual autocorrelations across repeated observations within GPs as recommended by Singer and Willett.^40(p85)^ Furthermore, the analyses were replicated in a separate 3-year sample of data, finding virtually identical results for all but 1 medication type. However, the study is not without limitations. It was not possible to assess the clinical appropriateness of prescribing decisions within individual encounters in this data set, and as such, any conclusions about the appropriateness of prescribing over the day are necessarily speculative. The order number of the patient encounter within a GP’s clinic day was used as a surrogate for decision fatigue in the present study. While this is accepted as standard in the literature, it is possible that other accumulating factors such as increasing levels of general fatigue, increasing stress levels, or running behind schedule and facing greater time pressure could contribute to the effects observed. Such factors would, if present, affect prescribing decisions in the same direction as decision fatigue, making an overestimation of the decision fatigue effect possible. A concern with all observational studies of decision fatigue is that case ordering or timing may be determined in advance (due to appointment scheduling) and as such is possibly influenced by unobservable factors correlated with outcomes. However, our modeling approach attempts to minimize the risk of confounding by including a multitude of control variables such as key characteristics of patients (age, sex, indigenous status, etc.), physicians (age, sex, rurality of practice, etc.), and encounters (weekday, season, and year). The data did not include detail on whether an encounter was prescheduled, same-day scheduled, or a walk-in appointment. As the acuteness of a patient visit could be correlated with it not being prescheduled, it would have been useful to control for this information if it were available in the data set. Due to high levels of missingness and recording inconsistencies across different waves of the BEACH study, we did not include new versus repeat prescriptions in the analyses. Theoretically, repeat prescriptions are expected to be less cognitively effortful than new prescriptions and therefore might be less/differently affected by decision fatigue effects. Future studies of decision fatigue and prescribing decisions should aim to account for distinction between new and repeat prescriptions where possible.

One final, and theoretically important, limitation is that the data used in this study lacked information on whether GPs took breaks over the course of their working day. Breaks would be expected to reset the decision fatigue effect for decisions made immediately after the break by interrupting the sequence of consecutive decisions (e.g., see Allan et al.^12^). As data on the timing of breaks was not present in the main BEACH data set, it was not possible to test this here. As there is evidence that office-based GPs rarely take a significant restorative break during their workdays,^21^ we would expect that if breaks occurred in this particular setting, they would be unlikely to be sufficient to significantly change our results.

In conclusion, in the present large, representative data set, antibiotic-prescribing rates increased significantly and statin and osteoporosis medication-prescribing rates decreased significantly over the working day with every additional patient encounter, in a pattern that is consistent with GPs’ becoming decision fatigued. Benzodiazepine-prescribing rates significantly decreased in the initial sample; however, this effect was not replicated in a second time period and was driven by a large dropoff in prescribing rates at the end of the shifts with high numbers of patient encounters. Consequently, we found no convincing evidence of decision fatigue effects in benzodiazepine prescribing. These findings extend the current literature and establish decision fatigue as a possible actionable target for optimizing prescribing. Limiting numbers of patient encounters completed without a break, adding automated prompts to the electronic health record designed to remind GPs to consider issuing preventive prescriptions where appropriate, and scheduling sufficient breaks might be approaches with the potential to support consistent patient care. Future studies should aim to uncover the mechanisms underpinning the decision fatigue effect and look to design and test interventions aiming to minimize negative effects of decision fatigue on health care provision.

## Supplemental Material

sj-docx-1-mdm-10.1177_0272989X241263823 – Supplemental material for Assessing Decision Fatigue in General Practitioners’ Prescribing Decisions Using the Australian BEACH Data Set Supplemental material, sj-docx-1-mdm-10.1177_0272989X241263823 for Assessing Decision Fatigue in General Practitioners’ Prescribing Decisions Using the Australian BEACH Data Set by Mona Maier, Daniel Powell, Christopher Harrison, Julie Gordon, Peter Murchie and Julia L. Allan in Medical Decision Making
