# Supplementary material for: Assessing Decision Fatigue in General Practitioners’ Prescribing Decisions Using the Australian BEACH Data Set
Source: Med Decis Making. 2024 Jul 26;44(6):627–40. doi: 10.1177/0272989X241263823 (PMC11346129; doi:10.1177/0272989X241263823)
Supplement: sj-docx-1-mdm-10.1177_0272989X241263823 – Supplemental material for Assessing Decision Fatigue in General Practitioners’ Prescribing Decisions Using the Australian BEACH Data Set [file sj-docx-1-mdm-10.1177_0272989X241263823.docx]

Assessing decision fatigue in general practitioners’ prescribing decisions using the Australian BEACH dataset

# Supplementary Materials

## S1: Medication categories and associated Anatomical Therapeutic Chemical classification codes

| **Medication Category** | **ATC codes** |
| --- | --- |
| Antibiotics | J01 |
| Opioids | N02A |
| Benzodiazepines | N05BA, N05CD |
| Statins | C10AA, C10BA, C10BX |
| Osteoporosis drugs | M05BA, M05BB, A12A, A11CC, M05BX, G03XC01, G03XC02, G03XC03, H05AA02 |

Supplementary Table 1: Medication categories and associated Anatomical Therapeutic Chemical (ATC) classification codes.

## S2: Detailed GLMM results

### S2.1: Antibiotics

| **Dependent variable: Antibiotics prescription**  **Recorded (1 = yes, 0 = no)** | **OR** | **2014-2016**  **95% CI**  **(SE)** | ***P* value** | **OR** | **2000-2003**  **95% CI**  **(SE)** | ***P* value** |
| --- | --- | --- | --- | --- | --- | --- |
| *Fixed effects ^a^:*     Intercept | 0.112 | 0.099-0.126 | <0.001 | 0.116 | 0.102-0.131 | <0.001 |
| Encounter’s ordinal position effect | 1.006 | 1.004-1.007 | <0.001 | 1.004 | 1.002-1.005 | <0.001 |
| GP Sex (ref. Male)  Female | 0.872 | 0.831-0.916 | <0.001 | 0.907 | 0.865-0.952 | <0.001 |
| GP Age group (ref. <45)  45-54  55+ | 0.995  1.025 | 0.937-1.056  0.968-1.086 | 0.863  0.392 | 1.035  1.219 | 0.982-1.091  1.151-1.290 | 0.198  <0.001 |
| Rurality (ref. Outer regional/remote)  Inner regional  Major City | 0.933  1.064 | 0.854-1.019  0.982-1.152 | 0.123  0.129 | 0.915  0.959 | 0.842-0.994  0.891-1.031 | 0.036  0.259 |
| Year (ref. 2016/2001)  2014  2015 | 0.989  1.058 | 0.935-1.047  1.001-1.118 | 0.710  0.048 | 1.156  1.096 | 1.096-1.220  1.039-1.157 | <0.001  <0.001 |
| Weekday (ref. Monday)  Tuesday  Wednesday  Thursday  Friday  Saturday  Sunday | 0.930  0.881  0.893  0.931  1.496  1.430 | 0.892-0.971  0.841-0.922  0.852-0.937  0.886-0.978  1.351-1.656  1.226-1.669 | <0.001  <0.001  <0.001  0.005  <0.001  <0.001 | 0.927  0.912  0.888  0.899  1.159  1.378 | 0.886-0.971  0.870-0.956  0.846-0.931  0.856-0.945  1.067-1.259  1.186-1.602 | 0.001  <0.001  <0.001  <0.001  <0.001  <0.001 |
| Season (ref. Winter)  Spring  Summer  Autumn | 0.869  0.814  0.807 | 0.817-0.924  0.762-0.868  0.760-0.858 | <0.001  <0.001  <0.001 | 0.913  0.735  0.803 | 0.861-0.968  0.690-0.783  0.757-0.852 | 0.003  <0.001  <0.001 |
| Patient Sex (ref. Male)  Female | 1.110 | 1.079-1.143 | <0.001 | 1.018 | 0.991-1.045 | 0.186 |
| Patient Age group (ref. 85+)  0-4  5-14  15-24  25-44  45-64  65-84 | 1.768  2.205  1.662  1.248  1.058  0.989 | 1.626-1.921  2.032-2.392  1.538-1.797  1.161-1.341  0.986-1.135  0.924-1.058 | <0.001  <0.001  <0.001  <0.001  0.115  0.742 | 2.369  2.832  1.998  1.406  1.11  0.921 | 2.154-2.605  2.577-3.112  1.825-2.187  1.288-1.535  1.018-1.210  0.845-1.004 | <0.001  <0.001  <0.001  <0.001  0.018  0.060 |
| Type of visit (ref. Seen previously)  New to practice | 1.185 | 1.121-1.253 | <0.001 | 1.158 | 1.108-1.212 | <0.001 |
| Health care/benefits card status (ref. Non-Holder)  Holder | 0.950 | 0.920-0.982 | 0.002 | 0.977 | 0.948-1.007 | 0.125 |
| Non-English-speaking background (ref. No)  Yes | 0.878 | 0.828-0.930 | <0.001 | 0.935 | 0.885-0.988 | 0.016 |
| Socio-Economic Index (ref. Low)  High Level of Advantage in Area | 1.043 | 1.005-1.084 | 0.028 | 1.037 | 1.000-1.075 | 0.048 |
| Indigenous status (ref. Non-indigenous)  Indigenous | 1.293 | 1.164-1.437 | <0.001 | 1.328 | 1.181-1.494 | <0.001 |
| *Random effects:*  *Level 2 (between-person):* |  |  |  |  |  |  |
| Variance in intercept | 0.504 | (0.017) | <0.001 | 0.504 | (0.016) | <0.001 |
| Variance in effect | 0.007 | (0.002) | <0.001 | 0.002 | (0.002) | <0.001 |
| Covariance | 0.003 | (0.001) | <0.001 | -0.003 | (0.001) | 0.015 |
| *Level 1 (within-person):*     Residual     Autocorrelation | 0.952  0.021 | (0.003)  (0.002) | <0.001  <0.001 | 0.952  0.021 | (0.003)  (0.002) | <0.001  <0.001 |
| *Note.*  *Fixed effects: Odds ratios;*  *Random effects: (Co-)variance parameter estimates; where covariance is N/A, this was omitted to facilitate model convergence; and residual estimates.* | | | | | | |

### S2.2: Opioids

| **Dependent variable: Opioids prescription**  **Recorded (1 = yes, 0 = no)** | **OR** | **2014-2016**  **95% CI**  **(SE)** | ***P* value** | **OR** | **2000-2003**  **95% CI**  **(SE)** | ***P* value** |
| --- | --- | --- | --- | --- | --- | --- |
| *Fixed effects ^a^:*     Intercept | 0.051 | 0.044-0.059 | <0.001 | 0.034 | 0.028-0.028 | <0.001 |
| Encounter’s ordinal position effect | 0.998 | 0.996-1.001 | 0.133 | 1.000 | 0.998-1.003 | 0.786 |
| GP Sex (ref. Male)  Female | 0.810 | 0.762-0.861 | <0.001 | 0.791 | 0.735-0.850 | <0.001 |
| GP Age group (ref. <45)  45-54  55+ | 1.010  1.052 | 0.935-1.090  0.980-1.128 | 0.807  0.160 | 1.087  1.030 | 1.007-1.173  0.948-1.120 | 0.033  0.486 |
| Rurality (ref. Outer regional/remote)  Inner regional  Major City | 0.995  0.803 | 0.899-1.102  0.728-0.886 | 0.928  <0.001 | 0.935  0.826 | 0.835-1.046  0.744-0.917 | 0.238  <0.001 |
| Year (ref. 2016/2003)  2014  2015 | 0.969  1.020 | 0.904-1.039  0.952-1.093 | 0.378  0.574 | 0.894  0.967 | 0.827-0.967  0.895-1.045 | 0.005  0.396 |
| Weekday (ref. Monday)  Tuesday  Wednesday  Thursday  Friday  Saturday  Sunday | 1.014  1.020  1.060  1.001  1.218  1.215 | 0.953-1.080  0.956-1.088  0.995-1.130  0.933-1.074  1.031-1.438  0.942-1.567 | 0.653  0.545  0.073  0.983  0.020  0.133 | 0.952  0.963  0.949  0.958  1.229  1.593 | 0.880-1.029  0.889-1.043  0.873-1.032  0.882-1.042  1.060-1.423  1.244-2.039 | 0.214  0.356  0.220  0.317  0.006  <0.001 |
| Season (ref. Winter)  Spring  Summer  Autumn | 1.012  0.9980.944 | 0.935-1.095  0.921-1.081  0.874-1.018 | 0.771  0.956  0.135 | 1.049  0.991  0.945 | 0.961-1.144  0.905-1.087  0.865-1.032 | 0.285  0.854  0.207 |
| Patient Sex (ref. Male)  Female | 0.963 | 0.924-1.004 | 0.075 | 0.879 | 0.837-0.923 | <0.001 |
| Patient Age group (ref. 85+)  0-4  5-14  15-24  25-44  45-64  65-84 | 0.002  0.028  0.399  1.043  1.396  0.858 | <0.001-0.011  0.017-0.045  0.347-0.459  0.949-1.147  1.282-1.520  0.793-0.928 | <0.001  <0.001  <0.001  0.383  <0.001  <0.001 | 0.012  0.073  0.711  1.809  1.694  1.071 | 0.005-0.027  0.051-0.104  0.604-0.837  1.575-2.079  1.480-1.938  0.936-1.225 | <0.001  <0.001  <0.001  <0.001  <0.001  0.320 |
| Type of visit (ref. Seen previously)  New to practice | 0.727 | 0.651-0.813 | <0.001 | 0.949 | 0.863-1.043 | 0.279 |
| Health care/benefits card status (ref. Non-Holder)  Holder | 2.376 | 2.256-2.502 | <0.001 | 1.921 | 1.820-2.027 | <0.001 |
| Non-English-speaking background (ref. No)  Yes | 0.671 | 0.611-0.737 | <0.001 | 0.684 | 0.614-0.762 | <0.001 |
| Socio-Economic Index (ref. Low)  High Level of Advantage in Area | 0.857 | 0.814-0.901 | <0.001 | 0.872 | 0.821-0.926 | <0.001 |
| Indigenous status (ref. Non-indigenous)  Indigenous | 1.427 | 1.229-1.658 | <0.001 | 1.066 | 0.858-1.325 | 0.562 |
| *Random effects:*  *Level 2 (between-person):* |  |  |  |  |  |  |
| Variance in intercept | 0.303 | (0.016) | <0.001 | 0.391 | (0.019) | <0.001 |
| Variance in effect | <0.001 | (0.000) | 0.404 | <0.001 | (0.000) | 0.144 |
| Covariance | N/A | (N/A) | N/A | N/A | (N/A) | N/A |
| *Level 1 (within-person):*     Residual     Autocorrelation | 0.901  0.008 | (0.003)  (0.008) | <0.001  <0.001 | 0.858  0.009 | (0.003)  0.002) | <0.001  <0.001 |
| *Note.*  *Fixed effects: Odds ratios;*  *Random effects: (Co-)variance parameter estimates; where covariance is N/A, this was omitted to facilitate model convergence; and residual estimates.* | | | | | | |

### S2.3: Benzodiazepines

| **Dependent variable: Benzodiazepine prescription**  **Recorded (1 = yes, 0 = no)** | **OR** | **2014-2016**  **95% CI**  **(SE)** | ***P* value** | **OR** | **2000-2003**  **95% CI**  **(SE)** | ***P* value** |
| --- | --- | --- | --- | --- | --- | --- |
| *Fixed effects ^a^:*     Intercept | 0.013 | 0.010-0.016 | <0.001 | 0.021 | 0.017-0.026 | <0.001 |
| Encounter’s ordinal position effect | 0.996 | 0.992-0.999 | 0.008 | 0.999 | 0.996-1.001 | 0.329 |
| GP Sex (ref. Male)  Female | 0.932 | 0.862-1.008 | 0.079 | 1.009 | 0.937-1.088 | 0.805 |
| GP Age group (ref. <45)  45-54  55+ | 1.073  1.254 | 0.971-1.187  1.142-1.378 | 0.166  <0.001 | 1.147  1.355 | 1.059-1.243  1.239-1.481 | <0.001  <0.001 |
| Rurality (ref. Outer regional/remote)  Inner regional  Major City | 1.029  1.114 | 0.888-1.193  0.969-1.281 | 0.702  0.130 | 1.265  1.259 | 1.116-1.434  1.120-1.415 | <0.001  <0.001 |
| Year (ref. 2016/2003)  2014/2000  2015/2001 | 1.075  1.053 | 0.982-1.177  0.960-1.155 | 0.118  0.272 | 1.136  1.025 | 1.046-1.234  0.942-1.115 | 0.002  0.564 |
| Weekday (ref. Monday)  Tuesday  Wednesday  Thursday  Friday  Saturday  Sunday | 1.057  1.0541.0830.986  0.876  0.902 | 0.975-1.146  0.968-1.149  0.997-1.177  0.898-1.083  0.691-1.112  0.634-1.283 | 0.175  0.225  0.059  0.765  0.277  0.566 | 1.162  1.121  1.151  1.091  0.999  1.217 | 1.068-1.263  1.027-1.222  1.055-1.255  0.993-1.198  0.845-1.183  0.890-1.665 | <0.001  0.010  0.002  0.071  0.995  0.219 |
| Season (ref. Winter)  Spring  Summer  Autumn | 0.997  1.008  0.937 | 0.896-1.111  0.903-1.125  0.846-1.038 | 0.963  0.891  0.212 | 0.968  0.998  0.899 | 0.881-1.063  0.904-1.101  0.819-0.987 | 0.490  0.968  0.026 |
| Patient Sex (ref. Male)  Female | 1.117 | 1.057-1.181 | <0.001 | 1.084 | 1.033-1.139 | 0.001 |
| Patient Age group (ref. 85+)  0-4  5-14  15-24  25-44  45-64  65-84 | 0  0.021  0.411  1.426  1.330  0.819 | <0.001-0.004  0.009-0.047  0.343-0.493  1.258-1.617  1.189-1.489  0.740-0.908 | <0.001  <0.001  <0.001  <0.001  <0.001  <0.001 | 0.004  0.020  0.272  0.820  0.881  0.913 | 0.001-0.013  0.011-0.034  0.229-0.323  0.724-0.929  0.787-0.987  0.821-1.016 | <0.001  <0.001  <0.001  0.002  0.029  0.096 |
| Type of visit (ref. Seen previously)  New to practice | 0.696 | 0.601-0.806 | <0.001 | 0.759 | 0.677-0.850 | <0.001 |
| Health care/benefits card status (ref. Non-Holder)  Holder | 2.605 | 2.427-2.795 | <0.001 | 2.190 | 2.056-2.333 | <0.001 |
| Non-English-speaking background (ref. No)  Yes | 0.488 | 0.428-0.557 | 0.716 | 0.554 | 0.489-0.628 | <0.001 |
| Socio-Economic Index (ref. Low)  High Level of Advantage in Area | 0.987 | 0.921-1.058 | <0.001 | 0.896 | 0.841-0.955 | <0.001 |
| Indigenous status (ref. Non-indigenous)  Indigenous | 1.385 | 1.113-1.722 | 0.004 | 1.342 | 1.047-1.719 | 0.020 |
| *Random effects:*  *Level 2 (between-person):* |  |  |  |  |  |  |
| Variance in intercept | 0.834 | (0.027) | <0.001 | 0.738 | (0.025) | <0.001 |
| Variance in effect | 0.025 | (0.002) | <0.001 | 0.017 | (0.002) | <0.001 |
| Covariance | -0.013 | (0.002) | N/A | -0.008 | (0.002) | <0.001 |
| *Level 1 (within-person):*     Residual     Autocorrelation | 0.752  0.005 | (0.002)  (0.002) | <0.001  0.018 | 0.863  0.010 | (0.003)  (0.002) | <0.001  <0.001 |
| *Note.*  *Fixed effects: Odds ratios;*  *Random effects: (Co-)variance parameter estimates; where covariance is N/A, this was omitted to facilitate model convergence; and residual estimates.* | | | | | | |

### S2.4: Statins

| **Dependent variable: Statins prescription**  **Recorded (1 = yes, 0 = no)** | **OR** | **2014-2016**  **95% CI**  **(SE)** | ***P* value** | **OR** | **2000-2003**  **95% CI**  **(SE)** | ***P* value** |
| --- | --- | --- | --- | --- | --- | --- |
| *Fixed effects ^a^:*     Intercept | 0.036 | 0.030-0.044 | <0.001 | 0.017 | 0.013-0.022 | <0.001 |
| Encounter’s ordinal position effect | 0.984 | 0.981-0.988 | <0.001 | 0.977 | 0.973-0.980 | <0.001 |
| GP Sex (ref. Male)  Female | 1.146 | 1.071-1.227 | <0.001 | 1.191 | 1.099-1.291 | <0.001 |
| GP Age group (ref. <45)  45-54  55+ | 1.289  1.345 | 1.181-1.407  1.239-1.460 | <0.001  <0.001 | 1.086  1.241 | 0.996-1.185  1.130-1.364 | 0.061  <0.001 |
| Rurality (ref. Outer regional/remote)  Inner regional  Major City | 0.969  1.043 | 0.857-1.096  0.929-1.170 | 0.617  0.474 | 0.966  1.005 | 0.846-1.102  0.893-1.130 | 0.604  0.940 |
| Year (ref. 2016/2003)  2014  2015 | 1.039  1.077 | 0.962-1.122  0.997-1.163 | 0.326  0.061 | 0.996  0.968 | 0.913-1.087  0.886-1.058 | 0.930  0.472 |
| Weekday (ref. Wednesday)  Monday  Tuesday  Thursday  Friday  Saturday  Sunday | 0.995  0.989  0.962  0.9690.790  0.713 | 0.918-1.078  0.918-1.065  0.888-1.042  0.890-1.055  0.633-0.987  0.426-1.192 | 0.907  0.763  0.343  0.469  0.038  0.197 | 0.994  1.007  0.972  0.901  0.872  0.723 | 0.899-1.100  0.928-1.092  0.892-1.060  0.819-0.992  0.700-1.085  0.435-1.204 | 0.912  0.876  0.519  0.034  0.218  0.213 |
| Season (ref. Winter)  Spring  Summer  Autumn | 1.114  1.096  1.116 | 1.015-1.223  0.999-1.202  1.022-1.218 | 0.023  0.052  0.014 | 0.967  1.047  0.941 | 0.872-1.071  0.946-1.159  0.851-1.040 | 0.513  0.370  0.233 |
| Patient Sex (ref. Male)  Female | 0.650 | 0.619-0.682 | <0.001 | 0.684 | 0.644-0.725 | <0.001 |
| Patient Age group (ref. 85+)  0-4  5-14  15-24  25-44  45-64  65-84 | 0.000  0.000  0.018  0.159  1.266  1.988 | <0.001->999.99  <0.001->999.99  0.010-0.033  0.135-0.186  1.138-1.409  1.798-2.198 | 0.700  0.196  <0.001  <0.001  <0.001  <0.001 | 0.019  0.022  0.041  0.392  3.008  3.808 | 0.007-0.053  0.008-0.060  0.022-0.077  0.308-0.499  2.437-3.712  3.096-4.683 | <0.001  <0.001  <0.001  <0.001  <0.001  <0.001 |
| Type of visit (ref. Seen previously)  New to practice | 0.637 | 0.539-0.754 | <0.001 | 0.524 | 0.430-0.640 | <0.001 |
| Health care/benefits card status (ref. Non-Holder)  Holder | 1.008 | 0.951-1.067 | 0.795 | 1.154 | 1.085-1.227 | <0.001 |
| Non-English-speaking background (ref. No)  Yes | 1.123 | 1.022-1.234 | 0.016 | 1.241 | 1.118-1.379 | <0.001 |
| Socio-Economic Index (ref. Low)  High Level of Advantage in Area | 0.952 | 0.899-1.009 | 0.099 | 0.970 | 0.899-1.045 | 0.421 |
| Indigenous status (ref. Non-indigenous)  Indigenous | 1.213 | 0.961-1.530 | 0.105 | 1.021 | 0.698-1.494 | 0.913 |
| *Random effects:*  *Level 2 (between-person):* |  |  |  |  |  |  |
| Variance in intercept | 0.309 | (0.018) | <0.001 | 0.371 | (0.023) | <0.001 |
| Variance in effect | 0.001 | (0.001) | <0.001 | <0.001 | (0.000) | <0.001 |
| Covariance | N/A | (N/A) | N/A | N/A | (N/A) | N/A |
| *Level 1 (within-person):*     Residual     Autocorrelation | 0.721  0.007 | (0.002)  (0.002) | <0.001  <0.001 | 0.770  0.004 | (0.002)  (0.002) | <0.001  0.059 |
| *Note.*  *Fixed effects: Odds ratios;*  *Random effects: (Co-)variance parameter estimates; where covariance is N/A, this was omitted to facilitate model convergence; and residual estimates.* | | | | | | |

### S2.5: Osteoporosis medications

| **Dependent variable: Osteoporosis medication prescription**  **Recorded (1 = yes, 0 = no)** | **OR** | **2014-2016**  **95% CI**  **(SE)** | ***P* value** | **OR** | **2000-2003**  **95% CI**  **(SE)** | ***P* value** |
| --- | --- | --- | --- | --- | --- | --- |
| *Fixed effects ^a^:*     Intercept | 0.009 | 0.007-0.012 | <0.001 | 0.003 | 0.002-0.005 | <0.001 |
| Encounter’s ordinal position effect | 0.981 | 0.976-0.986 | <0.001 | 0.989 | 0.983-0.995 | <0.001 |
| GP Sex (ref. Male)  Female | 1.646 | 1.483-1.826 | <0.001 | 1.507 | 1.328-1.710 | <0.001 |
| GP Age group (ref. <45)  45-54  55+ | 1.036  1.022 | 0.905-1.186  0.901-1.159 | 0.609  0.736 | 1.138  1.173 | 0.989-1.309  1.009-1.363 | 0.071  0.038 |
| Rurality (ref. Outer regional/remote)  Inner regional  Major City | 1.176  1.119 | 0.960-1.439  0.924-1.355 | 0.117  0.251 | 1.100  1.184 | 0.868-1.393  0.960-1.459 | 0.430  0.115 |
| Year (ref. 2016/2003)  2014  2015 | 1.033  0.913 | 0.917-1.164  0.808-1.031 | 0.591  0.143 | 0.748  0.770 | 0.652-0.859  0.669-0.887 | <0.001  <0.001 |
| Weekday (ref. Monday)  Tuesday  Wednesday  Thursday  Friday  Saturday  Sunday | 1.059  1.0211.0091.031  0.818  1.180 | 0.935-1.199  0.897-1.163  0.883-1.154  0.893-1.189  0.561-1.193  0.659-2.114 | 0.366  0.751  0.893  0.679  0.297  0.577 | 1.145  1.061  1.059  0.971  0.816  0.577 | 0.956-1.370  0.883-1.275  0.879-1.276  0.800-1.178  0.528-1.260  0.224-1.486 | 0.141  0.530  0.544  0.765  0.358  0.254 |
| Season (ref. Winter)  Spring  Summer  Autumn | 1.040  0.800  0.814 | 0.909-1.191  0.691-0.927  0.709-0.935 | 0.564  0.003  0.004 | 1.107  0.969  0.883 | 0.942-1.300  0.817-1.149  0.747-1.043 | 0.214  0.716  0.142 |
| Patient Sex (ref. Male)  Female | 2.805 | 2.525-3.116 | <0.001 | 4.464 | 3.801-5.241 | <0.001 |
| Patient Age group (ref. 85+)  0-4  5-14  15-24  25-44  45-64  65-84 | 0.017  0.052  0.124  0.213  0.354  0.814 | 0.008-0.038  0.029-0.094  0.093-0.166  0.179-0.255  0.305-0.410  0.722-0.918 | <0.001  <0.001  <0.001  <0.001  <0.001  <0.001 | 0.011  0.012  0.025  0.064  0.457  1.242 | 0.003-0.043  0.003-0.047  0.012-0.050  0.045-0.089  0.369-0.566  1.025-1.505 | <0.001  <0.001  <0.001  <0.001  <0.001  0.027 |
| Type of visit (ref. Seen previously)  New to practice | 0.391 | 0.277-0.552 | <0.001 | 0.596 | 0.422-0.841 | 0.003 |
| Health care/benefits card status (ref. Non-Holder)  Holder | 1.091 | 0.992-1.200 | 0.072 | 1.253 | 1.104-1.422 | <0.001 |
| Non-English-speaking background (ref. No)  Yes | 1.723 | 1.499-1.980 | <0.001 | 1.084 | 0.893-1.317 | 0.413 |
| Socio-Economic Index (ref. Low)  High Level of Advantage in Area | 0.976 | 0.879-1.084 | 0.648 | 1.038 | 0.913-1.180 | 0.567 |
| Indigenous status (ref. Non-indigenous)  Indigenous | 0.617 | 0.371-1.028 | 0.064 | 0.461 | 0.176-1.210 | 0.116 |
| *Random effects:*  *Level 2 (between-person):* |  |  |  |  |  |  |
| Variance in intercept | 1.192 | (0.036) | <0.001 | 0.837 | (0.052) | <0.001 |
| Variance in effect | 0.057 | (0.002) | <0.001 | N/A | (N/A) | N/A |
| Covariance | -0.048 | (0.004) | <0.001 | N/A | (N/A) | N/A |
| *Level 1 (within-person):*     Residual     Autocorrelation | 0.530  <0.001 | (0.002)  (0.002) | <0.001  0.923 | 0.636  <0.001 | (0.001)  (0.002) | <0.001  0.909 |
| *Note.*  *Fixed effects: Odds ratios;*  *Random effects: (Co-)variance parameter estimates; where covariance is N/A, this was omitted to facilitate model convergence; and residual estimates.* | | | | | | |

## S3: Conditional logistic regression results

The analysis approach uses conditional logistic regression models as described by Allison (2006) for logistic models for three or more observations per person [41]. Conditional logistic regression is a standard approach for analysing panel data, and it is used when subjects are measured at multiple time points. In our data, subjects (General Practitioners) were measured at each patient encounter over the course of a shift. Using conditional logistic regression with fixed effects by GP, our results can be interpreted as “within group” (i.e., GP-specific) effects.

### S3.1: Antibiotics

| **Dependent variable: Antibiotics prescription**  **Recorded (1 = yes, 0 = no)** | **OR** | **2014-2016**  **95% CI** |
| --- | --- | --- |
| Encounter’s ordinal position effect | 1.005 | 1.003-1.007 |
| Patient Sex (ref. Male)  Female | 1.114 | 1.084-1.146 |
| Patient Age group (ref. 85+)  0-4  5-14  15-24  25-44  45-64  65-84 | 1.701  2.132  1.605  1.211  1.038  0.979 | 1.572-1.841  1.970-2.306  1.487-1.731  1.130-1.298  0.970-1.110  0.917-1.045 |
| Type of visit (ref. Seen previously)  New to practice | 1.195 | 1.135-1.259 |
| Health care/benefits card status (ref. Non-Holder)  Holder | 0.962 | 0.931-0.994 |
| Non-English-speaking background (ref. No)  Yes | 0.863 | 0.812-0.918 |
| Socio-Economic Index (ref. Low)  High Level of Advantage in Area | 1.020 | 0.977-1.065 |
| Indigenous status (ref. Non-indigenous)  Indigenous | 1.238 | 1.109-1.381 |
| Weekday (ref. Wednesday)  Monday  Tuesday  Thursday  Friday  Saturday  Sunday | 1.141  1.064  1.023  1.052  1.685  1.598 | 1.091-1.193  1.020-1.109  0.977-1.070  1.002-1.105  1.540-1.844  1.373-1.860 |
| Season (ref. Winter)  Spring  Summer  Autumn | 1.014  0.861  0.819 | 0.841-1.221  0.700-1.059  0.679-0.987 |

S3.2: Opioids

| **Dependent variable: Opioids prescription**  **Recorded (1 = yes, 0 = no)** | **OR** | **2014-2016**  **95% CI** |
| --- | --- | --- |
| Encounter’s ordinal position effect | 0.998 | 0.995-1.000 |
| Patient Sex (ref. Male)  Female | 0.971 | 0.933-1.010 |
| Patient Age group (ref. 85+)  0-4  5-14  15-24  25-44  45-64  65-84 | 0.002  0.028  0.404  1.061  1.406  0.873 | <0.001-0.011  0.017-0.046  0.355-0.460  0.975-1.155  1.302-1.519  0.810-0.940 |
| Type of visit (ref. Seen previously)  New to practice | 0.707 | 0.634-0.788 |
| Health care/benefits card status (ref. Non-Holder)  Holder | 2.281 | 2.176-2.392 |
| Non-English-speaking background (ref. No)  Yes | 0.704 | 0.638-0.777 |
| Socio-Economic Index (ref. Low)  High Level of Advantage in Area | 0.883 | 0.831-0.938 |
| Indigenous status (ref. Non-indigenous)  Indigenous | 1.438 | 1.248-1.656 |
| Weekday (ref. Wednesday)  Monday  Tuesday  Thursday  Friday  Saturday  Sunday | 0.976  0.987  1.020  0.971  1.077  1.064 | 0.915-1.041  0.930-1.048  0.958-1.087  0.905-1.041  0.921-1.261  0.813-1.392 |
| Season (ref. Winter)  Spring  Summer  Autumn | 0.958  1.042  1.191 | 0.728-1.261  0.772-1.407  0.909-1.560 |

S3.3: Benzodiazepines

| **Dependent variable: Benzodiazepines prescription**  **Recorded (1 = yes, 0 = no)** | **OR** | **2014-2016**  **95% CI** |
| --- | --- | --- |
| Encounter’s ordinal position effect | 0.995 | 0.992-0.999 |
| Patient Sex (ref. Male)  Female | 1.123 | 1.064-1.184 |
| Patient Age group (ref. 85+)  0-4  5-14  15-24  25-44  45-64  65-84 | <0.001  0.021  0.417  1.397  1.293  0.820 | <0.001->999.9  0.009-0.047  0.350-0.496  1.253-1.557  1.168-1.431  0.743-0.905 |
| Type of visit (ref. Seen previously)  New to practice | 0.715 | 0.618-0.827 |
| Health care/benefits card status (ref. Non-Holder)  Holder | 2.505 | 2.353-2.667 |
| Non-English-speaking background (ref. No)  Yes | 0.491 | 0.429-0.563 |
| Socio-Economic Index (ref. Low)  High Level of Advantage in Area | 0.934 | 0.863-1.010 |
| Indigenous status (ref. Non-indigenous)  Indigenous | 1.535 | 1.266-1.861 |
| Weekday (ref. Wednesday)  Monday  Tuesday  Thursday  Friday  Saturday  Sunday | 0.976  0.987  1.020  0.971  1.077  1.064 | 0.915-1.041  0.930-1.048  0.958-1.087  0.905-1.041  0.921-1.261  0.813-1.392 |
| Season (ref. Winter)  Spring  Summer  Autumn | 1.209  1.247  1.298 | 0.840-1.739  0.840-1.853  0.895-1.881 |

S3.4: Statins

| **Dependent variable: Statins prescription**  **Recorded (1 = yes, 0 = no)** | **OR** | **2014-2016**  **95% CI** |
| --- | --- | --- |
| Encounter’s ordinal position effect | 0.989 | 0.986-0.992 |
| Patient Sex (ref. Male)  Female | 0.646 | 0.616-0.678 |
| Patient Age group (ref. 85+)  0-4  5-14  15-24  25-44  45-64  65-84 | <0.001  <0.001  0.019  0.166  1.287  1.979 | <0.001->999.9  <0.001->999.9  0.010-0.035  0.142-0.194  1.160-1.427  1.797-2.180 |
| Type of visit (ref. Seen previously)  New to practice | 0.694 | 0.590-0.816 |
| Health care/benefits card status (ref. Non-Holder)  Holder | 1.006 | 0.950-1.066 |
| Non-English-speaking background (ref. No)  Yes | 1.106 | 0.996-1.227 |
| Socio-Economic Index (ref. Low)  High Level of Advantage in Area | 0.959 | 0.889-1.034 |
| Indigenous status (ref. Non-indigenous)  Indigenous | 1.168 | 0.917-1.489 |
| Weekday (ref. Wednesday)  Monday  Tuesday  Thursday  Friday  Saturday  Sunday | 0.979  0.975  0.947  0.966  0.775  0.756 | 0.905-1.058  0.907-1.047  0.876-1.023  0.888-1.051  0.619-0.970  0.492-1.161 |
| Season (ref. Winter)  Spring  Summer  Autumn | 1.169  1.437  1.279 | 0.834-1.639  0.976-2.115  0.889-1.839 |

S3.5: Osteoporosis medications

| **Dependent variable: Osteoporosis medication prescription**  **Recorded (1 = yes, 0 = no)** | **OR** | **2014-2016**  **95% CI** |
| --- | --- | --- |
| Encounter’s ordinal position effect | 0.988 | 0.983-0.993 |
| Patient Sex (ref. Male)  Female | 2.721 | 2.464-3.005 |
| Patient Age group (ref. 85+)  0-4  5-14  15-24  25-44  45-64  65-84 | 0.018  0.057  0.135  0.227  0.376  0.839 | 0.008-0.041  0.034-0.095  0.102-0.179  0.192-0.268  0.326-0.432  0.746-0.944 |
| Type of visit (ref. Seen previously)  New to practice | 0.401 | 0.293-0.549 |
| Health care/benefits card status (ref. Non-Holder)  Holder | 1.086 | 0.984-1.197 |
| Non-English-speaking background (ref. No)  Yes | 1.637 | 1.409-1.902 |
| Socio-Economic Index (ref. Low)  High Level of Advantage in Area | 0.940 | 0.827-1.069 |
| Indigenous status (ref. Non-indigenous)  Indigenous | 0.640 | 0.382-1.070 |
| Weekday (ref. Wednesday)  Monday  Tuesday  Thursday  Friday  Saturday  Sunday | 0.980  1.047  0.997  1.042  0.734  1.333 | 0.860-1.115  0.928-1.182  0.877-1.134  0.906-1.199  0.505-1.067  0.678-2.618 |
| Season (ref. Winter)  Spring  Summer  Autumn | 1.018  1.139  1.107 | 0.623-1.663  0.646-2.008  0.648-1.889 |

## S4: Conditional logistic regression results examining possible non-linear effects

The models presented in S3 were rerun using dummy variables reflecting groups of encounters as categories (1-5, 6-10, 11-15, etc.) instead of the linear encounter’s ordinal position effect that was estimated above. All other predictor variables were kept the same.

### S4.1: Antibiotics

| **Dependent variable: Antibiotics prescription**  **Recorded (1 = yes, 0 = no)** | **OR** | **2014-2016**  **95% CI** |
| --- | --- | --- |
| Encounter’s ordinal position effect (ref. 1-5)  6-10  11-15  16-20  21-25  26-30  31-35  36-40  41+ | 1.134  1.134  1.152  1.115  1.106  1.175  1.135  1.250 | 1.093-1.176  1.091-1.179  1.102-1.205  1.055-1.178  1.030-1.188  1.064-1.298  0.983-1.310  1.065-1.468 |
| Patient Sex (ref. Male)  Female | 1.113 | 1.082-1.144 |
| Patient Age group (ref. 85+)  0-4  5-14  15-24  25-44  45-64  65-84 | 1.701  2.140  1.608  1.214  1.041  0.980 | 1.572-1.841  1.978-2.315  1.490-1.734  1.133-1.301  0.973-1.114  0.918-1.046 |
| Type of visit (ref. Seen previously)  New to practice | 1.194 | 1.134-1.257 |
| Health care/benefits card status (ref. Non-Holder)  Holder | 0.959 | 0.928-0.991 |
| Non-English-speaking background (ref. No)  Yes | 0.863 | 0.811-0.917 |
| Socio-Economic Index (ref. Low)  High Level of Advantage in Area | 1.019 | 0.976-1.064 |
| Indigenous status (ref. Non-indigenous)  Indigenous | 1.234 | 1.106-1.377 |
| Weekday (ref. Wednesday)  Monday  Tuesday  Thursday  Friday  Saturday  Sunday | 1.143  1.065  1.023  1.051  1.682  1.597 | 1.092-1.195  1.021-1.110  0.977-1.070  1.001-1.104  1.537-1.841  1.372-1.859 |
| Season (ref. Winter)  Spring  Summer  Autumn | 1.014  0.861  0.819 | 0.842-1.222  0.700-1.059  0.680-0.987 |

S4.2: Opioids

| **Dependent variable: Opioids prescription**  **Recorded (1 = yes, 0 = no)** | **OR** | **2014-2016**  **95% CI** |
| --- | --- | --- |
| Encounter’s ordinal position effect (ref. 1-5)  6-10  11-15  16-20  21-25  26-30  31-35  36-40  41+ | 1.023  1.039  0.919  0.950  0.874  0.900  1.130  0.963 | 0.972-1.077  0.984-1.097  0.862-0.981  0.877-1.029  0.784-0.974  0.769-1.053  0.917-1.394  0.744-1.246 |
| Patient Sex (ref. Male)  Female | 0.970 | 0.933-1.009 |
| Patient Age group (ref. 85+)  0-4  5-14  15-24  25-44  45-64  65-84 | 0.002  0.028  0.405  1.063  1.408  0.872 | <0.001-0.011  0.017-0.046  0.356-0.461  0.976-1.156  1.304-1.521  0.810-0.939 |
| Type of visit (ref. Seen previously)  New to practice | 0.706 | 0.633-0.787 |
| Health care/benefits card status (ref. Non-Holder)  Holder | 2.278 | 2.173-2.388 |
| Non-English-speaking background (ref. No)  Yes | 0.704 | 0.638-0.777 |
| Socio-Economic Index (ref. Low)  High Level of Advantage in Area | 0.883 | 0.832-0.938 |
| Indigenous status (ref. Non-indigenous)  Indigenous | 1.436 | 1.246-1.654 |
| Weekday (ref. Wednesday)  Monday  Tuesday  Thursday  Friday  Saturday  Sunday | 0.977  0.988  1.020  0.969  1.072  1.064 | 0.915-1.042  0.931-1.049  0.958-1.087  0.904-1.040  0.916-1.255  0.814-1.392 |
| Season (ref. Winter)  Spring  Summer  Autumn | 0.959  1.042  1.190 | 0.729-1.262  0.772-1.407  0.909-1.559 |

S4.3: Benzodiazepines

| **Dependent variable: Benzodiazepines prescription**  **Recorded (1 = yes, 0 = no)** | **OR** | **2014-2016**  **95% CI** |
| --- | --- | --- |
| Encounter’s ordinal position effect (ref. 1-5)  6-10  11-15  16-20  21-25  26-30  31-35  36-40  41+ | 1.020  1.007  0.971  0.987  0.824  0.953  0.777  0.615 | 0.954-1.091  0.937-1.083  0.892-1.057  0.888-1.096  0.709-0.958  0.777-1.168  0.564-1.072  0.400-0.947 |
| Patient Sex (ref. Male)  Female | 1.121 | 1.063-1.182 |
| Patient Age group (ref. 85+)  0-4  5-14  15-24  25-44  45-64  65-84 | <0.001  0.021  0.416  1.396  1.294  0.820 | <0.001->999.999  0.009-0.047  0.349-0.496  1.253-1.556  1.169-1.433  0.743-0.905 |
| Type of visit (ref. Seen previously)  New to practice | 0.714 | 0.617-0.825 |
| Health care/benefits card status (ref. Non-Holder)  Holder | 2.501 | 2.349-2.663 |
| Non-English-speaking background (ref. No)  Yes | 0.491 | 0.428-0.562 |
| Socio-Economic Index (ref. Low)  High Level of Advantage in Area | 0.933 | 0.863-1.009 |
| Indigenous status (ref. Non-indigenous)  Indigenous | 1.534 | 1.265-1.860 |
| Weekday (ref. Wednesday)  Monday  Tuesday  Thursday  Friday  Saturday  Sunday | 0.960  1.020  1.050  0.943  0.867  0.876 | 0.883-1.044  0.944-1.103  0.966-1.141  0.860-1.034  0.691-1.089  0.575-1.335 |
| Season (ref. Winter)  Spring  Summer  Autumn | 1.298  1.210  1.248 | 0.896-1.882  0.841-1.742  0.840-1.853 |

S4.4: Statins

| **Dependent variable: Statins prescription**  **Recorded (1 = yes, 0 = no)** | **OR** | **2014-2016**  **95% CI** |
| --- | --- | --- |
| Encounter’s ordinal position effect (ref. 1-5)  6-10  11-15  16-20  21-25  26-30  31-35  36-40  41+ | 0.869  0.819  0.843  0.748  0.744  0.777  0.755  0.679 | 0.818-0.924  0.767-0.875  0.780-0.910  0.675-0.828  0.649-0.854  0.638-0.946  0.570-1.002  0.476-0.968 |
| Patient Sex (ref. Male)  Female | 0.648 | 0.617-0.679 |
| Patient Age group (ref. 85+)  0-4  5-14  15-24  25-44  45-64  65-84 | <0.001  <0.001  0.019  0.165  1.283  1.976 | <0.001->999.999  <0.001->999.999  0.010-0.034  0.141-0.193  1.156-1.423  1.794-2.176 |
| Type of visit (ref. Seen previously)  New to practice | 0.696 | 0.592-0.819 |
| Health care/benefits card status (ref. Non-Holder)  Holder | 1.009 | 0.953-1.069 |
| Non-English-speaking background (ref. No)  Yes | 1.107 | 0.997-1.229 |
| Socio-Economic Index (ref. Low)  High Level of Advantage in Area | 0.959 | 0.889-1.034 |
| Indigenous status (ref. Non-indigenous)  Indigenous | 1.171 | 0.919-1.492 |
| Weekday (ref. Wednesday)  Monday  Tuesday  Thursday  Friday  Saturday  Sunday | 0.976  0.973  0.947  0.966  0.776  0.757 | 0.903-1.055  0.906-1.045  0.876-1.023  0.888-1.051  0.620-0.972  0.493-1.163 |
| Season (ref. Winter)  Spring  Summer  Autumn | 1.169  1.438  1.281 | 0.834-1.639  0.977-2.117  0.891-1.843 |

S4.5: Osteoporosis medications

| **Dependent variable: Osteoporosis medication prescription**  **Recorded (1 = yes, 0 = no)** | **OR** | **2014-2016**  **95% CI** |
| --- | --- | --- |
| Encounter’s ordinal position effect (ref. 1-5)  6-10  11-15  16-20  21-25  26-30  31-35  36-40  41+ | 0.938  0.873  0.786  0.837  0.928  0.579  0.647  0.384 | 0.849-1.035  0.783-0.973  0.688-0.899  0.705-0.994  0.741-1.162  0.383-0.875  0.366-1.144  0.166-0.886 |
| Patient Sex (ref. Male)  Female | 2.722 | 2.465-3.006 |
| Patient Age group (ref. 85+)  0-4  5-14  15-24  25-44  45-64  65-84 | 0.018  0.056  0.135  0.226  0.375  0.838 | 0.008-0.040  0.033-0.095  0.102-0.178  0.191-0.268  0.326-0.431  0.745-0.943 |
| Type of visit (ref. Seen previously)  New to practice | 0.401 | 0.293- 0.550 |
| Health care/benefits card status (ref. Non-Holder)  Holder | 1.086 | 0.984-1.197 |
| Non-English-speaking background (ref. No)  Yes | 1.639 | 1.411- 1.904 |
| Socio-Economic Index (ref. Low)  High Level of Advantage in Area | 0.942 | 0.829-1.071 |
| Indigenous status (ref. Non-indigenous)  Indigenous | 0.642 | 0.384-1.074 |
| Weekday (ref. Wednesday)  Monday  Tuesday  Thursday  Friday  Saturday  Sunday | 0.979  1.047  0.997  1.044  0.735  1.335 | 0.860-1.115  0.927-1.182  0.877-1.133  0.907-1.201  0.505-1.069  0.680-2.623 |
| Season (ref. Winter)  Spring  Summer  Autumn | 1.107  1.018  1.141 | 0.648-1.888  0.623-1.663  0.647-2.011 |
